# Trans-Zeatin Enhances *Auxenochlorella pyrenoidosa* Growth by Coordinating Carbon–Nitrogen Metabolism and Antioxidant Defense

**DOI:** 10.3390/microorganisms13112554

**Published:** 2025-11-08

**Authors:** Yong-Lan Ma, Min Li, Qian Lei, Hai-Jun Ma, Ya-Jing An

**Affiliations:** 1 School of Biological Science and Engineering, North Minzu University, Yinchuan 750021, China; is_maylwwh@yeah.net (Y.-L.M.); leiqian1971@126.com (Q.L.); mahaijun_118@163.com (H.-J.M.); yajing_imu@163.com (Y.-J.A.); 2 Ningxia Grape & Wine Innovation Center, North Minzu University, Yinchuan 750021, China

**Keywords:** *Auxenochlorella pyrenoidosa*, trans-zeatin, growth regulation, mechanism

## Abstract

*Auxenochlorella pyrenoidosa*, a promising edible bioresource, can be efficiently and safely cultivated using exogenous phytohormones to enhance its productivity. This study employed multi-omics analysis to systematically investigate the effects and mechanisms of exogenous trans-Zeatin (tZ) on the growth and metabolism of *A. pyrenoidosa*. Results demonstrated that 10 mg/L tZ significantly promoted algal growth, increasing biomass by 166 ± 3.35% at 72 hours (h), while concurrently elevating cellular soluble protein (SP), carbohydrate (CHO), and chlorophyll a (Chla) content. tZ also strengthened the antioxidant defense system, evidenced by reduced reactive oxygen species (ROS) levels, enhanced activities of antioxidant enzymes (superoxide dismutase (SOD) and catalase (CAT)), upregulation of glutathione metabolism, and decreased lipid peroxidation product (malondialdehyde (MDA)). Furthermore, tZ activated key metabolic pathways, including nitrogen metabolism, photosynthetic carbon fixation, and porphyrin biosynthesis, leading to the accumulation of arginine and polyamines, etc. This study reveals that tZ promotes microalgal growth by coordinately regulating carbon–nitrogen metabolic networks and antioxidant systems, providing a theoretical foundation for phytohormone-augmented microalgae cultivation technologies.

## 1. Introduction

Food security, energy crisis, and climate change represent major challenges to the sustainable development of human society. As the most important primary producers in Earth’s aquatic ecosystems, microalgae have emerged as a highly promising sustainable resource due to their efficient carbon fixation via photosynthesis, minimal requirement for arable land, and ability to synthesize a wide range of high-value products through their metabolic processes. In addition, microalgae exhibit metabolic diversity, capable of autotrophic, heterotrophic, and mixotrophic growth. This versatility allows them to adapt to various cultivation environments, including the use of wastewater or other non-potable water sources, thereby significantly reducing freshwater consumption [1,2,3].

Microalgae demonstrate broad application potential in fields such as food, energy, environment, and pharmaceuticals [4,5]. The microalgae industry can directly contribute to multiple United Nations Sustainable Development Goals (SDGs) by integrating aquaculture feed, high-value compound extraction, bioenergy production, and wastewater treatment [6]. Improving the productivity of microalgae is not only key to enhancing economic benefits but also central to amplifying its environmental and social impacts.

In order to improve the production efficiency of microalgae, researchers have employed various strategies. One common approach involves stress factors, such as high light intensity and nitrogen starvation, which induce the synthesis and accumulation of high-value compounds, often at the cost of biomass production [7,8]. However, this growth-inhibiting model is not suitable for production systems focused on maximizing biomass (e.g., for protein or whole biomass production). Consequently, the development of safe, efficient, and low-cost growth promoters remains a major research priority in microalgae biotechnology [7,9,10]. Among various strategies, plant hormones have emerged as a highly attractive growth promoter due to their efficiency and environmental friendliness, making them a significant research focus.

Phytohormones are trace natural organic compounds produced within plants that regulate cell division, growth and development, as well as responses to environmental stresses [11]. It is now widely recognized that the production of these signaling molecules is not exclusive to higher plants. Bacteria (such as *Azospirillum*, *Bacillus*, *Pseudomonas*) and fungi (such as *Fusarium*, *Trichoderma*) are also capable of producing various hormones, including auxins, cytokinins, gibberellins, ethylene, and abscisic acid [12,13]. Due to their high efficacy and safety, phytohormones have emerged as an attractive alternative strategy for enhancing microalgal growth and biomass quality. Existing studies have revealed species-specific variations in the responses of microalgae to phytohormones. Moreover, the receptors and signaling pathways of phytohormones in microalgae differ significantly from those in higher plants, and may involve unique, yet unidentified mechanisms [9]. Therefore, further investigation into the growth-regulating effects of phytohormones on specific microalgal species is essential to optimize their application.

Trans-zeatin (tZ), a cytokinin first isolated from immature maize seeds in 1963 [14], was named zeatin based on its origin. As a naturally occurring adenine-type cytokinin, tZ is renowned for its crucial role in promoting cell division and regulating plant growth and development [15]. It promotes cell division and influences other aspects of the cell cycle, including inhibiting chlorophyll and protein degradation, reducing respiration rates, and maintaining cell viability [16].

Importantly, tZ plays a key role in the regulation of stress tolerance in microalgae. Studies have shown that tZ can alleviate oxidative damage caused by cadmium stress in *Dunaliella armata* by enhancing the antioxidant system [17]. Similarly, tZ can improve the tolerance of *Acutodesmus obliquus* to lead stress by regulating hormone homeostasis and mediating detoxification through thiols [18]. Furthermore, tZ has been shown to enhance photosynthetic performance, biomass yield, and lipid productivity in *Acutodesmus obliquus* under nitrogen stress [19]. Notably, *Chlorella* sp. achieves its maximum growth rate at only 0.1 mM tZ, a concentration ten times lower than that required for indole-3-acetic acid (IAA), gibberellic acid (GA), or abscisic acid (ABA), demonstrating a significantly higher sensitivity specifically to tZ [20].These characteristics of tZ present new potential for developing microalgal cultivation processes with higher economic output and lower operational risks.

*Auxenochlorella pyrenoidosa*, a species rich in essential amino acids, vitamins, and other nutrients [21], has been approved as a novel food ingredient by regulatory agencies including the U.S. FDA and China’s National Health Commission [22]. Despite the significant potential of tZ in regulating microalgal growth and metabolism, current understanding of the growth-regulatory mechanisms of tZ in *A. pyrenoidosa* remains limited and fragmentary. Key questions concerning the specific physiological effects of tZ and its underlying gene regulatory networks in this species still lack. We hypothesize that exogenous tZ can have a positive effect on the growth and metabolite accumulation in *A. pyrenoidosa*. To address these gaps, this study adopts an integrated physiology-biochemistry and multi-omics approach to elucidate the regulatory mechanisms of exogenous tZ on the growth and metabolism of *A. pyrenoidosa*. The results are expected to establish a theoretical foundation and potential targets for developing tZ-enhanced cultivation strategies aimed at improving microalgal production.

## 2. Materials and Methods

### 2.1. Materials

*A. pyrenoidosa* with strain No. of FACHB-5 was purchased from the Institute of Hydrobiology, Chinese Academy of Sciences (Wuhan, China). tZ, with CAS No. of 1637-39-4 and HPLC grade, was purchased from Aladdin Biochemical Technology Co., Ltd. (Shanghai, China). Other chemicals used for microalgal culture medium preparation were purchased from Sinopharm Chemical Reagent Co., Ltd. (Shanghai, China).

### 2.2. Determination of A. pyrenoidosa Biomass

*A. pyrenoidosa* was pre-cultivated in Blue-Green 11 (BG-11) medium. The algal cultures were incubated in an orbital shaker under the following conditions: temperature (25 ± 1) °C, light intensity 4000 lux, a light/dark cycle of 12:12 h, and agitation speed of 150 r/min.

Algal cells in the exponential growth phase were transferred into 250 mL conical flasks. The cell density was adjusted with BG-11 medium to an initial optical density at 680 nm (*OD*_680_) of approximately 0.035–0.040, equivalent to a cell density of 1 × 10^5^ cells/mL. Exogenous tZ was added at concentrations of 0, 5, 10, and 20 mg/L. The group without tZ addition (0 mg/L tZ) was designated as the control (CK) group. All treated groups were cultivated in a static light incubator under the same temperature, light, and photoperiod conditions as the pre-culture. To prevent algal cell sedimentation, the flasks were manually shaken three times daily [23].

Algal cell density was monitored every 24 h by measuring *OD*_680_ using a UV spectrophotometer with model G-9S from Feile Instrument Co., Ltd. (Nanjing, China). The change in biomass was expressed as a percentage relative to the CK group according to the following formula: ρ_Treatment_/ρ_CK_ × 100%.

### 2.3. Determination of Biochemical Indexes of A. pyrenoidosa

20 mL of algal liquid was taken and centrifuged at 8000× *g* for 10 min, the supernatant was discarded, and the precipitated algal cells were resuspended in phosphate buffer (PBS, 0.01 mol/L, pH = 7.8), and then the cells were broken by ultrasonic wave in ice bath (power 300 w, ultrasonic wave 3 s, interval 7 s, total time 15 min) [24]. The broken liquid was centrifuged, and the supernatant was taken for the subsequent determination of biochemical indexes.

The biochemical indices, including soluble protein (SP), total antioxidant capacity (TAC), catalase (CAT), malondialdehyde (MDA), reactive oxygen species (ROS), superoxide dismutase (SOD), were measured using kits from Suzhou Keming Co., Ltd. (Suzhou, China). The corresponding catalog numbers are BCAP-1-W, FRAP-1-G, CAT-1-W, MDA-1-Y, ROS-1-Y, SOD-1-W. The assays were performed according to the manufacturer’s instructions.

Chlorophyll a (Chla) was extracted using 80% acetone and stored in the dark at 4 °C for 24 h. The algal suspension was then centrifuged at 8000× *g* for 5 min. The absorbance values of the extract were measured at 646 nm and 663 nm using a UV-Vis spectrophotometer. The Chla was calculated using the following equation:

Chla (mg/L) = 12.2 × A_663_ − 2.81 × A_646_ (1)

The carbohydrate (CHO) content was determined using the phenol–sulfuric acid method. A 1 mL aliquot of algal culture was centrifuged at 8000× *g* for 10 min. The pellet was resuspended in 2 mL of deionized water. To this, 1 mL of 6% phenol and 5 mL of concentrated sulfuric acid were added, and the mixture was heated in a boiling water bath for 15 min. The optical density was then measured at 490 nm, and the CHO content was calculated from the standard curve [25].

### 2.4. Selection of Omics Analysis Conditions

Based on the growth dynamics and key biochemical indicators obtained under different tZ concentrations as presented in Section 2.2 and Section 2.3 and following the principle of selecting conditions that result in the most significant biomass accumulation and the most sensitive physiological responses, the optimal tZ treatment conditions for transcriptomic and metabolomic analysis were selected.

### 2.5. Transcriptomic Analysis

Transcriptomic analysis was performed on algal cells treated with 10 mg/L tZ for 72 h, three biological replicates were performed for each treatment group. A 40 mL aliquot of algal culture was collected and centrifuged at 4 °C and 8000× *g* for 10 min. The supernatant was discarded, and the algal cell pellet was resuspended in 4 mL of high-purity water. The suspension was vortexed and centrifuged again. This process was repeated three times. The final algal cell pellet was transferred to a 2 mL centrifuge tube and stored at −80 °C for subsequent use.

Total RNA was extracted using TRIzol reagent (Invitrogen, Carlsbad, CA, USA), and genomic DNA was removed by treatment with DNase I Takara Bio (TaKara Bio, Shiga, Japan). RNA integrity was assessed using agarose gel electrophoresis, and the quality was determined with an Agilent 5300 Bioanalyzer (Agilent Technologies, Santa Clara, CA, USA). RNA quantification was performed using a Qubit 4.0 Fluorometer (Thermo Fisher Scientific, Waltham, MA, USA).

RNA purification, reverse transcription, library preparation, and sequencing were conducted at Shanghai Majorbio Bio-pharm Technology Co., Ltd. (Shanghai, China) following the manufacturer’s protocols (Illumina, San Diego, CA, USA). For library construction, mRNA was isolated using the oligo(dT) method, and the mRNA was fragmented. Reverse transcription was carried out using the SuperScript Double-Stranded cDNA Synthesis Kit (Invitrogen) to synthesize cDNA. The synthesized cDNA was then subjected to terminal repair, phosphorylation, and ‘A’ base addition. The target cDNA fragments were selected for PCR amplification and enrichment. Finally, sequencing was performed using the NovaSeq X Plus/DNBSEQ-T7 sequencer. (Illumina, San Diego, CA, USA; BGI Genomics Co., Ltd., Shenzhen, China).

The KEGG database (https://www.kegg.jp/kegg/pathway.html, accessed on 11 July 2025) was employed for gene classification, enabling KEGG annotation of differentially expressed genes (DEGs). KEGG pathway enrichment analysis was conducted using the Python (version 3.9) scipy package (https://scipy.org/install/, accessed on 16 July 2025) with Fisher’s exact test employed for the calculations.

### 2.6. Metabolomic Analysis

The sample preparation for metabolomics was consistent with that for transcriptomics, while six biological replicates were performed for each treatment group. Metabolomic analysis was conducted in collaboration with Shanghai Majorbio Bio-pharm Technology Co., Ltd.

Algal cells were transferred to a 1.5 mL centrifuge tube, and 400 μL of extraction solvent (acetonitrile:methanol = 1:1, *v*:*v*) containing 0.02 mg/mL of internal standard (L-2-chlorophenylalanine) was added. The mixture was vortexed for 30 s and then subjected to low-temperature ultrasonic extraction for 30 min (5 °C, 40 KHz). After the extraction, the metabolites were allowed to stand at −20 °C for 30 min, followed by centrifugation at 4 °C, 8000× *g* for 15 min. The supernatant was collected, dried under nitrogen, and then re-dissolved in 100 μL of acetonitrile:water (1:1, *v*:*v*) solution. The mixture was subjected to low-temperature ultrasonic extraction for 5 min (5 °C, 40 KHz) and centrifuged again. The supernatant was transferred to a sample vial for subsequent analysis.

The samples were analyzed by LC-MS/MS using a high-resolution ultra-high-performance liquid chromatography coupled with a quadrupole-exactive high-field mass spectrometer (UHPLC-Q Exactive HF-X, Thermo Fisher Scientific, Waltham, MA, USA). Chromatographic conditions: 3 μL of the sample was injected onto an HSS T3 column (100 mm × 2.1 mm, 1.8 µm), and separation was followed by mass spectrometry detection. Mobile phase A consisted of 95% water and 5% acetonitrile (containing 0.1% formic acid), and mobile phase B consisted of 47.5% acetonitrile, 47.5% isopropanol, and 5% water (containing 0.1% formic acid). The flow rate was 0.40 mL/min, and the column temperature was maintained at 40 °C. The samples were analyzed in both positive and negative ion scan modes of mass spectrometry, with the mass scan range set to 70–1050 *m*/*z*. The sheath gas flow rate was 50 psi, auxiliary gas flow rate was 13 psi, and the auxiliary gas heating temperature was 425 °C. The capillary temperature was set to 325 °C. The positive mode ion spray voltage was set to 3500 V, and the negative mode ion spray voltage was set to −3500 V. The normalized collision energy was set to a cycle of 20–40–60 eV.

After analysis, the raw LC-MS data were imported into the metabolomics software Progenesis QI (version 3.0, Waters Corporation, Milford, MA, USA) for pre-analysis. The pre-processed data matrix was subjected to principal component analysis (PCA) and orthogonal partial least squares discriminant analysis (OPLS-DA) using the R package (version 1.6.2). Metabolites with VIP > 1 and *p* < 0.05 were considered differentially expressed metabolites (DEMs). Pathway enrichment analysis was performed using the Python (version 3.9) scipy package (https://scipy.org/install/, accessed on 16 July 2025) within the stats module.

### 2.7. Statistical Analysis

In this study, the results of all experiments are presented as the mean ± standard deviation (SD). Physiological and biochemical data were analyzed using one-way analysis of variance (ANOVA), with a significance threshold set at *p* < 0.05. The R software (version 3.5.1) (https://www.r-project.org/, accessed on 16 July 2025) was used to perform PCA [26]. The Simca (version 14.1) was used to perform OPLS-DA. The OriginPro 2021B Beta software facilitated the creation of volcano plots, heatmaps, bubble charts, and other graphical representations [27]. Additionally, transcriptional and metabolic data analysis was carried out on the Majorbio Cloud Platform (https://www.majorbio.com/, accessed on 21 August 2025). The correlation between transcriptional and metabolic data was assessed using OPLS-DA and the Pearson correlation algorithm.

## 3. Results

### 3.1. The Growth Improvement of tZ to A. pyrenoidosa

**Figure 1:**
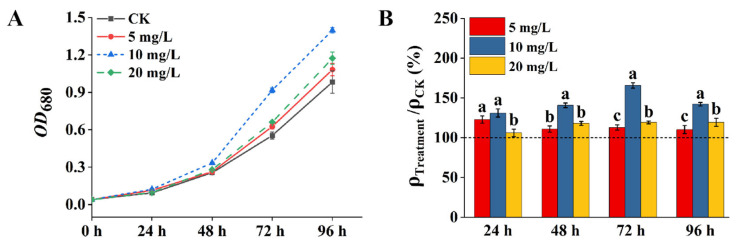
The effect of tZ in regulating the biomass of *A. pyrenoidosa*: (**A**) changes of absorbance values of algal cell solution at 680 nm; (**B**) the improvement of tZ to the biomass of *A. pyrenoidosa*. Note: Different lowercase letters above the bars indicate statistically significant differences among different tZ concentrations at the same time point, as determined by one-way ANOVA followed by Tukey’s test (*p* < 0.05).

The regulatory effect of tZ on the growth of *A. pyrenoidosa* is shown in Figure 1. The increase in optical density at 680 nm (*OD*_680_), indicating biomass accumulation, was most pronounced in the group treated with 10 mg/L tZ (Figure 1A). Furthermore, to quantitatively analyze the growth dynamics, linear regression was applied to the growth curves of the 10 mg/L tZ treatment group at different time intervals (0–24 h, 24–48 h, 48–72 h, 72–96 h). The calculated slopes of the growth curves for these intervals were 0.003, 0.008, 0.024, and 0.020, respectively, indicating that the 10 mg/L tZ treatment group exhibited the fastest growth during the 48–72 h period.

The differences in the growth-promoting effects of tZ at various concentrations on *A. pyrenoidosa* were assessed using the ratio of ρ_Treatment_/ρ_CK_, as shown in Figure 1B. All concentrations of tZ promoted algal cell growth. After 48 h of cultivation, the growth promotion efficiency at 10 mg/L tZ was significantly higher than that observed in the 5 and 20 mg/L treatment groups. After 72 h, the growth promotion efficiency at 10 mg/L tZ reached its peak, with the ratio of ρ_Treatment_/ρ_CK_ of 166 ± 3.35%. Throughout the entire cultivation period, the growth promotion efficiency at 10 mg/L tZ exhibited a reversed U-shaped trend.

### 3.2. Influence of tZ on the Biochemical Characteristics of A. pyrenoidosa

**Figure 2:**
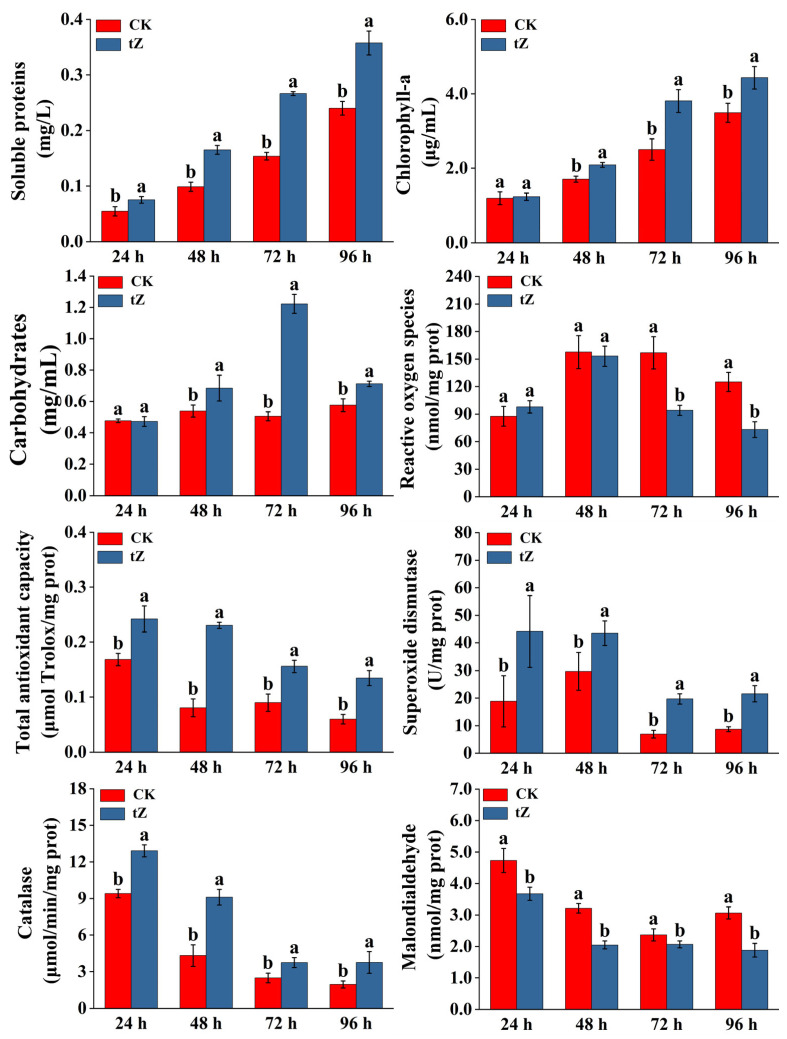
Effects of 10 mg/L tZ on biochemical characteristics for *A. pyrenoidosa.* Note: Different lowercase letters above the bars indicate statistically significant differences among different tZ concentrations at the same time point, as determined by one-way ANOVA followed by Tukey’s test (*p* < 0.05).

The regulatory effect of tZ on the biochemical characteristics of *A. pyrenoidosa* is shown in Figure 2. Throughout the cultivation period, the SP in the 10 mg/L tZ treatment group was consistently significantly higher than that in the CK group (*p* < 0.05). At 72 h, the difference was the greatest, with the SP content in the treatment group being 1.73 times that of the CK group. This suggests that 10 mg/L tZ can promote the accumulation of SP in *A. pyrenoidosa* and enhance the metabolic activity of the algal cells.

After treatment with 10 mg/L tZ, significant differences in Chla content were observed between the treatment and CK groups during the later stages of cultivation (48–96 h) (*p* < 0.05). The treatment group exhibited 1.22, 1.52, and 1.27 times the Chla content of the CK group at 48, 72, and 96 h, respectively. Similarly, from 48 to 96 h, the CHO content in the tZ group increased significantly and was higher than that in the CK group (*p* < 0.05). The CHO content peaked at 72 h, reaching 1.22 ± 0.06 mg/mL.

Following treatment with 10 mg/L tZ, there was no significant difference in ROS levels between the treatment and CK groups from 24 to 48 h. However, during the 72–96 h cultivation period, ROS levels in the treatment group were significantly lower than those in the CK group (*p* < 0.05). Throughout the entire cultivation period, the TAC in the treatment group was significantly higher than that in the CK group (*p* < 0.05). Additionally, the activities of SOD and CAT in the treatment group were significantly higher than those in the CK group, while the MDA content in the treatment group was significantly lower than that in the CK group (*p* < 0.05).

### 3.3. Influence of tZ on the Gene Expression of A. pyrenoidosa

**Figure 3:**
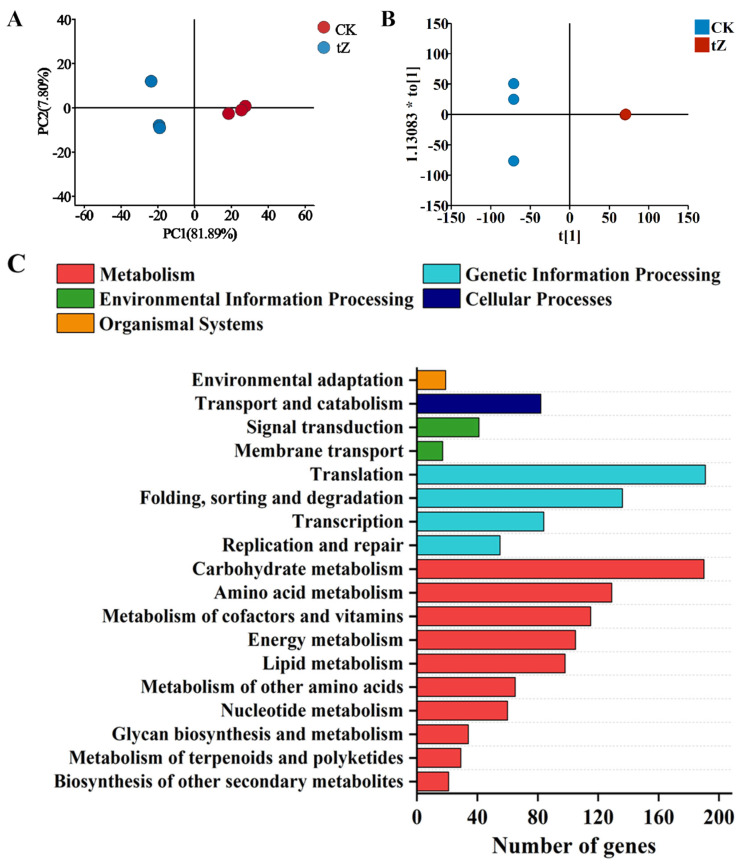

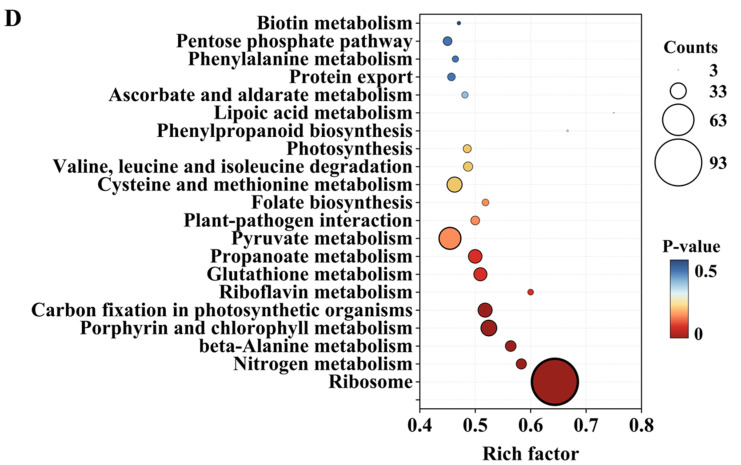
Transcriptomic analysis of *A. pyrenoidosa* after 72 h exposure to 10 mg/L tZ: (**A**) PCA; (**B**) OPLS-DA analysis; (**C**) KEGG functional annotation of DEGs; (**D**) KEGG pathway enrichment analysis of DEGs.

The transcriptomic analysis showed that the clean data of all samples with a Q30 base percentage greater than 94.24%, indicating good sequencing quality and a suitable reference genome (Table S1). PCA results are shown in Figure 3A, where the first two principal components (PC1 = 81.89%, PC2 = 7.8%) collectively explained 89.69% of the variance, with the CK and tZ treatment groups clearly separated along the PC1 axis. The results of OPLS-DA are presented in Figure 3B, with R^2^Y = 1 and Q^2^ = 0.997. The permutation test (Number of permutation tests = 200, *p* < 0.001) was significant, further confirming the differences between the CK and tZ treatment groups. A total of 2410 DEGs were identified based on |log_2_(FC)| ≥ 1 and *p* < 0.05, with 1167 upregulated and 1243 downregulated genes. The volcano plot and hierarchical clustering analysis of the DEGs are shown in Figure S1A,B.

KEGG functional annotation analysis of the DEGs is presented in Figure 3C, which revealed that the DEGs were primarily enriched in metabolic pathways, including carbohydrate metabolism, amino acid metabolism, metabolism of cofactors and vitamins, energy metabolism, and lipid metabolism. The number of DEGs enriched in these pathways were 190, 129, 115, 105, and 98, respectively.

KEGG pathway enrichment analysis of the DEGs is shown in Figure 3D. Significantly enriched metabolic pathways (*p* < 0.05) included the ribosome, nitrogen metabolism, beta-alanine metabolism, porphyrin and chlorophyll metabolism, and carbon fixation in photosynthetic organisms. The number of DEGs enriched in these pathways were 94, 21, 22, 32, and 29, respectively.

### 3.4. Influence of tZ on the Metabolites Expression of A. pyrenoidosa

**Figure 4:**
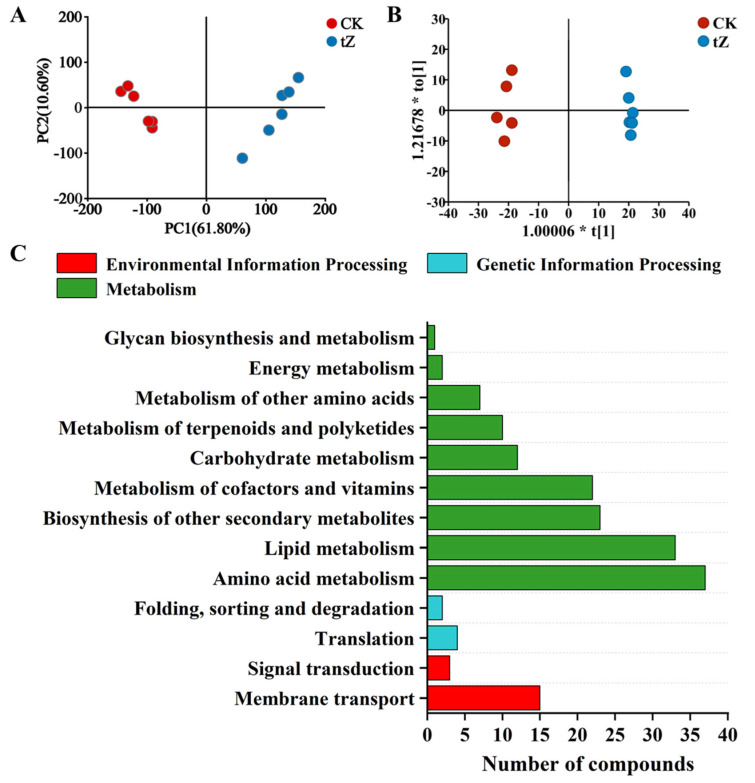

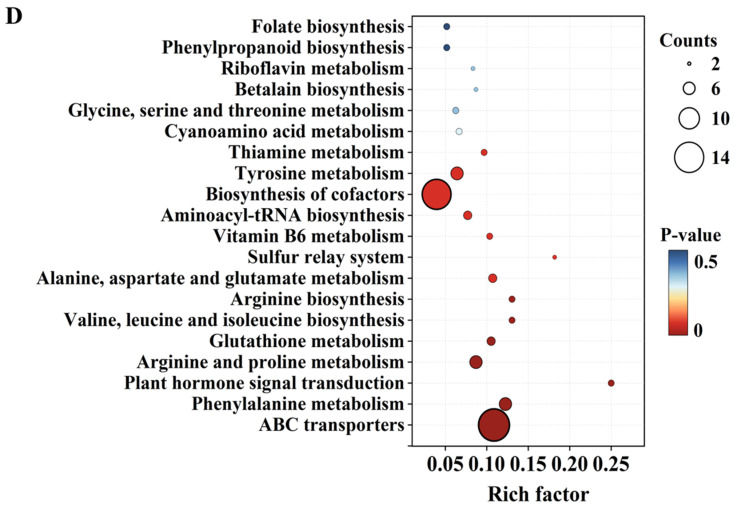
Metabolomic analysis of *A. pyrenoidosa* after 72 h exposure to 10 mg/L tZ: (**A**) PCA; (**B**) OPLS-DA analysis; (**C**) KEGG functional annotation of DEMs; (**D**) KEGG pathway enrichment analysis of DEMs.

The results of the metabolomic PCA and OPLS-DA analyses are shown in Figure 4A,B. The first two principal components in the PCA explained a cumulative 72.4% of the variance, with the CK and tZ treatment groups partially separated along the PC1 axis. The OPLS-DA analysis yielded R^2^Y = 0.995 and Q^2^ = 0.979, and the result of permutation test was significant (Number of permutation tests = 200, *p* < 0.001), further confirming the inter-group differences. Based on VIP > 1 and |log FC| ≥ 1, a total of 588 DEMs were identified in the algal cells, including 319 upregulated and 269 downregulated metabolites. The volcano plot and clustering analysis of the differential metabolites are shown in Figure S2A,B.

KEGG pathway annotation analysis of all DEMs is presented in Figure 4C. The pathways involved in metabolism were most enriched, including amino acid metabolism, lipid metabolism, biosynthesis of other secondary metabolites, metabolism of cofactors and vitamins, and carbohydrate metabolism, which are consistent with the transcriptomic results. Analysis of the categories of DEMs revealed that amino acid metabolism underwent significant changes, representing 47.92% of the differential metabolites. Additionally, amino acids and their derivatives, carbohydrates and their derivatives, and nucleotides and their derivatives also showed significant changes, accounting for 26.39%, 13.19%, and 11.81% of all differential metabolites, respectively (Figure S3).

The KEGG pathway enrichment analysis of the differential metabolites is shown in Figure 4D. Significantly enriched pathways (*p* < 0.05) included ABC transporter, phenylalanine metabolism, glutathione metabolism, arginine and proline metabolism, biosynthesis of cofactors, tyrosine metabolism, etc.

## 4. Discussion

### 4.1. The Effect of tZ on the Growth of A. pyrenoidosa: A Dose-Dependent Response

The results of this study showed that 10 mg/L tZ had the most pronounced promoting effect on the growth of *A. pyrenoidosa*, increasing the biomass by 166% after 72 h. Although the 20 mg/L tZ treatment group still exhibited higher biomass than the CK group, its promoting effect was significantly weaker than that in the 10 mg/L tZ group. This pattern is consistent with the hormesis phenomenon observed for various plant hormones in regulating plant growth and development [9], which refers to a biphasic response where low doses stimulate biological functions while high doses exert inhibitory effects [28,29].

In addition to promoting biomass accumulation, 10 mg/L tZ enhanced the accumulation of high-value metabolites such as proteins and fatty acids. The effects of other tZ concentrations on intracellular metabolite profiles, however, remain unclear. For instance, 20 mg/L tZ might have acted as a mild stressor that triggered the synthesis of certain metabolites. In biotechnological processes aimed at obtaining specific microalgal metabolites, the volumetric productivity of the target compound is a more meaningful indicator than biomass alone. Hence, further studies are warranted to systematically characterize the metabolomic responses of *A. pyrenoidosa* to different tZ concentrations and to assess their impacts on the production of specific high-value compounds.

### 4.2. The Effect of tZ on the Redox Equilibrium of A. pyrenoidosa

The maintenance of cellular redox homeostasis relies on the precise regulation of ROS levels, antioxidant enzyme activities, and redox signaling, which are essential for physiological functions such as cell proliferation, apoptosis, and energy metabolism. Plant hormones, as key regulatory factors, play a central role in regulating ROS metabolism and the antioxidant enzyme system in microalgae [30,31,32].This study found that tZ, as an important member of the cytokinin family, significantly enhances the antioxidant defense capacity of *A. pyrenoidosa*, primarily manifested by a decrease in ROS levels, an increase in antioxidant enzyme (SOD, CAT) activities, enhancement of the glutathione metabolic pathway, and a reduction in lipid peroxidation product MDA.

The intracellular ROS levels directly affect the redox status of cells. tZ’s regulation of ROS levels in *A. pyrenoidosa* cells exhibits a clear temporal pattern. Although there was no significant difference in ROS levels between the tZ treatment and CK groups from 24 to 48 h, a significant decrease was observed between 72 and 96 h (*p* < 0.05) in the tZ treatment group. This delayed effect suggests that tZ may act through indirect pathways, such as inducing antioxidant enzyme gene expression, rather than through direct ROS scavenging mechanisms.

Monitoring of SOD and CAT activities showed that tZ provides early protection by rapidly activating the antioxidant enzyme system. Experimental data indicated that the 10 mg/L tZ treatment group exhibited significantly higher SOD and CAT activities throughout the entire culture period compared to the CK group (*p* < 0.05). Notably, plant hormones have a universal regulatory effect on the antioxidant system in microalgae. For instance, IAA, indole-3-butyric acid (IBA), and phenylacetic acid can rapidly activate the enzymatic antioxidant system (such as ascorbate peroxidase (APX), CAT, SOD) in *Chlorella vulgaris*, thereby inhibiting lipid peroxidation and hydrogen peroxide accumulation [33]. IAA can alleviate atrazine-induced stress in *Cylindrospermum stagnale* by enhancing SOD, CAT, and APX activities [34]. Zeatin can enhance the antioxidant system of the *Dunaliella armata* to reduce cadmium-induced oxidative damage [17]. Furthermore, studies show that zeatin activates the Keap-1/HO-1 signaling pathway, thereby enhancing the antioxidant capacity of the receptor and improving its stress resistance [35]. This study confirms that zeatin plays a key oxidative defense role in *A. pyrenoidosa* by rapidly inducing antioxidant enzyme activity, with its regulatory effect similar to that of the aforementioned plant hormones.

**Figure 5:**
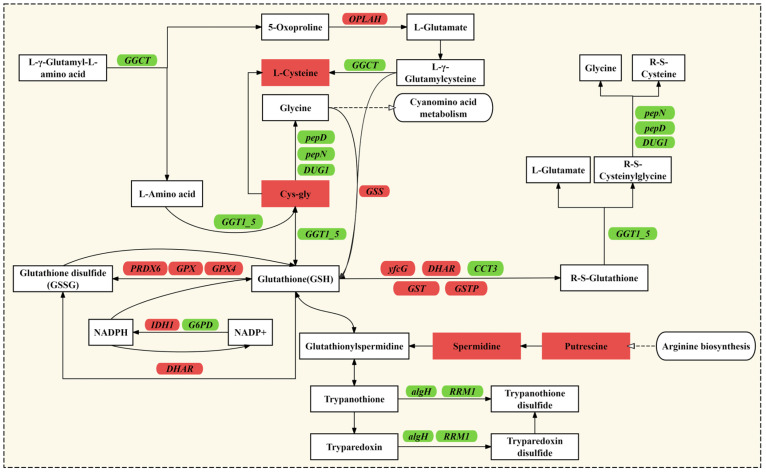
Enrichment analysis of DEGs and DEMs in the glutathione metabolism pathway of *A. pyrenoidosa* following treatment with 10 mg/L tZ. Note: In the pathway diagram, metabolites are represented by rectangular boxes, and genes are denoted by rounded-edge boxes. Red and green colors indicate upregulation and downregulation, respectively.

GSH, as the primary reducing molecule inside cells, is involved in the removal of ROS, maintaining protein reduction status, and regulating signal transduction. Transcriptomic and metabolomic analyses showed that the glutathione pathway in *A. pyrenoidosa* was significantly enriched under tZ induction, as shown in Figure 5. L-cysteine is a key precursor for GSH synthesis, and its increased content may directly promote GSH synthesis [36]. Additionally, Cys-Gly, an intermediate product of GSH degradation, accumulates, which may indicate an accelerated GSH turnover [37]. Polyamines such as putrescine and spermidine have been shown to synergize with GSH to reduce oxidative damage [38,39]. Moreover, the expression of 11 key enzymes, including Glutathione synthetase (*GSS*), Glutathione peroxidase (*GPX*), Glutathione S-transferases (*GST*), Peroxiredoxin 6 (*PRDX6*), and 5-oxoprolinase, ATP-hydrolyzing (*OPLAH*), was enhanced. *GSS*, *GPX*, and *GST* are directly involved in GSH synthesis and detoxification, while *PRDX6* can reduce hydrogen peroxide (H_2_O_2_) to water, thus reducing oxidative stress damage to cells [40]. tZ enhances the ROS scavenging ability of *A. pyrenoidosa* by upregulating the expression of these key metabolites and enzymes, thereby maintaining the internal environment stability more effectively.

Additionally, in the glutathione metabolic pathway, 10 genes, such as Glucose-6-phosphate dehydrogenase (*G6PD*), Putative transcriptional regulator (*algH*), and T-complex protein 1 subunit gamma (*CCT3*), were found to be downregulated. We speculate that the downregulation of these genes may be related to the cellular stress response induced by tZ. For example, *G6PD* is an important enzyme in the glycolytic pathway, and its downregulation may make the cell more dependent on glutathione metabolism and other antioxidant mechanisms to cope with environmental stress [41].

TAC reflects the overall levels of various antioxidant macromolecules, small molecules, and enzymes in cells, providing a more comprehensive measure of the cell’s ability to combat free radicals and oxidative stress. MDA is a typical product of lipid peroxidation and can reflect the integrity of the cell membrane structure and the extent of oxidative damage. Throughout the entire culture period, the TAC in the tZ treatment group was significantly higher than that in the CK group, and the MDA content was significantly lower than that of the CK group (*p* < 0.05). These results together confirm that tZ not only enhances the enzymatic defense system but also maintains the integrity of the cell membrane and redox balance comprehensively.

Previous studies have shown that cytokinins can improve plant stress resistance and delay cellular aging by activating antioxidant enzymes, increasing the levels of natural antioxidants such as phenolic acids and flavonoids, and reducing ROS production [42,43]. The findings of this study align with previous research.

### 4.3. The Effect of tZ on the Photosynthesis and Carbon Metabolism of A. pyrenoidosa

In the later stages of cultivation (48–96 h), the tZ treated *A. pyrenoidosa* cells showed significantly higher Chla and CHO contents compared to the CK group. This result suggests that tZ may enhance photosynthetic efficiency by promoting chlorophyll biosynthesis, thereby driving CHO accumulation. This finding is consistent with previous research: cytokinins have been shown to regulate chloroplast development and help algae cope with environmental stress by modulating key factors of photosynthesis [18,44,45].

Further mechanistic studies revealed that tZ treatment significantly enriched the porphyrin metabolism pathway. As the core pathway for chlorophyll biosynthesis, porphyrin metabolism begins with the synthesis of 5-amino-4-oxovaleric acid (ALA) and undergoes multiple enzymatic reactions to ultimately produce chlorophyll. Transcriptomic data showed significant changes in the expression of key genes involved in porphyrin synthesis in the treatment group: the expressions of Heme synthase (*HCAR*) and UDP-glucuronosyltransferase (*UGT*) were enhanced. *HCAR* encodes heme synthase, which catalyzes the terminal conversion of protoheme to chlorophyll. Its upregulation helps accelerate porphyrin synthesis, thereby promoting chlorophyll accumulation [46,47]. The *UGT* gene plays a role in the transformation and regulation during porphyrin synthesis and may be associated with the stability and metabolism of chlorophyll. Its upregulation may contribute to promoting photosynthesis-related metabolic reactions [48].

Additionally, we observed a decrease in the expression of several key genes related to porphyrin degradation and transformation, such as Heme oxygenase (*HEPH*), Siroheme biosynthesis (*sirB*), and Chlorophyllide a oxygenase (*CAO*). The upregulation and downregulation cooperative regulatory patterns not only accelerate the biosynthesis of porphyrin into chlorophyll but also inhibit the degradation and conversion of chlorophyll precursors, promoting the accumulation of Chla.

Metabolomic analysis revealed an upregulation of urobilinogen levels and a downregulation of chlorophyllide a and pheophorbide a levels in the porphyrin metabolism pathway. These changes may be related to the regulation of chlorophyll synthesis and degradation processes [19].

It is noteworthy that transcriptomic analysis also revealed a significant enrichment of the carbon fixation in photosynthetic organisms’ pathway. Under tZ induction, the expression of genes encoding 17 key enzymes was enhanced, such as Malate dehydrogenase (*MDH2*), Transketolase (*tktA*), Phosphoglycerate kinase (*PGK*), Ribulose-phosphate 3-epimerase (*rpe*), Triose-phosphate isomerase (*TPI*), and Glyceraldehyde 3-phosphate dehydrogenase (*GAPDH*). *MDH2* is involved in a key step of the tricarboxylic acid (TCA) cycle, and its upregulation helps enhance energy production under aerobic conditions [49]. *tktA* and *rpe* are involved in the pentose phosphate pathway [50], which is a crucial step in carbon fixation during photosynthesis. Their upregulation helps the cell generate pentose sugars and promotes Nicotinamide Adenine Dinucleotide Phosphate Reduced (NADPH) synthesis. The upregulation of *PGK*, *GAPDH*, and *TPI* helps optimize the glycolysis and gluconeogenesis pathways [51]. These genes are involved in key steps of carbon metabolism, suggesting that tZ may promote carbon fixation and carbohydrate synthesis by regulating the expression of these genes.

The molecular-level changes mentioned above suggest that tZ may regulate multiple targets, including enhancing light energy capture efficiency, improving carbon assimilation capacity, and optimizing carbohydrate turnover, thereby collaboratively promoting the accumulation of photosynthetic products in *A. pyrenoidosa*.

### 4.4. The Effect of tZ on the Nitrogen Metabolism of A. pyrenoidosa

After treating *A. pyrenoidosa* with 10 mg/L tZ, the SP content of the cells was significantly increased, and nitrogen metabolism-related pathways were notably enriched. The expression of genes encoding several key enzymes involved in nitrogen metabolism was enhanced, such as Glutamate dehydrogenase (NAD(P)+) (*GLUD1_2*), Carbamate kinase (*arcC*), Glutamine synthetase (*GLUL*), Nitrate reductase (NAD(P)H) (*NR*), Hydroxylamine reductase (*hcp*), Cyanate lyase (*cynS*), and Fungal nitric oxide reductase (*CYP55*). These enzymes play a crucial role in regulating nitrogen metabolism. Among them, the upregulation of *GLUD1_2* promoted the conversion of glutamate to α-ketoglutarate, a process that is central to nitrogen assimilation and detoxification [52]. Meanwhile, the enhanced expression of *arcC* may increase the cell’s ability to uptake nitrogen sources [53], and the upregulation of *GLUL* strengthened the glutamine synthesis pathway, providing an important carrier for nitrogen transport and storage [54,55]. Notably, the upregulation of *NR* directly reflects enhanced nitrate assimilation capacity, as this enzyme catalyzes the reduction of nitrate to nitrite, which is the rate-limiting step in nitrate assimilation [56]. The coordinated upregulation of these genes forms a comprehensive regulatory network, from nitrogen source uptake (*arcC*), nitrate reduction (*NR*), to activation of the glutamate metabolism hub (*GLUD1_2* and *GLUL*), collectively promoting the nitrogen metabolism efficiency of *A. pyrenoidosa*.

Metabolomic analysis showed that tZ treatment significantly altered the levels of various nitrogen metabolism-related metabolites in *A. pyrenoidosa*. Among these, the levels of polyamines putrescine and spermidine were significantly upregulated. These compounds play crucial roles in plant and algal growth, cell division, and stress responses [57]. The increase in their content indicates that tZ enhances the cell’s nitrogen utilization efficiency by regulating polyamine metabolism, which aligns with the ability of polyamines to modulate nitrogen assimilation enzyme activity and amino acid synthesis pathways [58,59]. The levels of L-arginine and citrulline were also significantly elevated. These two amino acids play central roles in nitrogen metabolism, with arginine not only serving as an important nitrogen source but also participating in nitrogen metabolism regulation through the urea cycle [60]. Their accumulation suggests that tZ enhances nitrogen source uptake and assimilation by activating the arginine metabolism pathway. Additionally, the levels of branched-chain amino acid metabolic intermediates, such as 3-methyl-2-oxopentanoic acid, 3-isopropylmalic acid, and 2-isopropylmalic acid, as well as other organic acids, were significantly increased. This suggests that tZ may optimize nitrogen assimilation efficiency and carbon metabolism balance by regulating branched-chain amino acid metabolism and the TCA cycle [61]. The coordinated changes in these metabolites together form a complex metabolic regulatory network, reflecting the multi-level regulatory role of tZ in nitrogen metabolism in *A. pyrenoidosa*.

Previous studies have confirmed that the supplementation of tZ can stimulate the activity of key factors and enzymes involved in nitrogen metabolism, thereby promoting cell growth and division [16]. The above molecular-level results further demonstrate that tZ enhances the expression of multiple nitrogen metabolism-related genes, promoting the synthesis of nitrogen metabolism-related metabolites. The synergistic interaction between these genes and metabolites helps algal cells improve the uptake, assimilation, and conversion efficiency of nitrogen sources, thus enhancing their growth and metabolic activity [62].

### 4.5. Real-World Applicability

From a technical application perspective, this study demonstrates that 10 mg/L tZ effectively enhances both biomass accumulation and the accumulation of high-value metabolites in *A. pyrenoidosa*, providing theoretical support for industrial-scale cultivation. Moreover, while promoting algal cell growth, tZ also enhances the antioxidant defense capacity of the cells. It is hypothesized that tZ supplementation could simultaneously improve algal cells’ adaptability to environmental fluctuations such as light and temperature, thereby increasing the stability and stress resistance of the entire cultivation system.

The use of safe growth regulators in microalgae cultivation is crucial, as microalgae not only have important applications in fields such as food and feed but are also closely related to the ecological environment. tZ is widely present in edible plants, and its natural origin provides assurance for its application in microalgae cultivation.

From an economic standpoint, although the addition of tZ incurs some material costs, its significant enhancement of biomass presents a core potential for economic benefits. The future cost–benefit ratio of this technology will primarily depend on the actual procurement cost of tZ as a bulk reagent, as well as the product value increase driven by biomass growth.

## 5. Conclusions

This study integrates multi-omics and physiological–biochemical techniques to reveal the multiple mechanisms by which exogenous tZ promotes the growth of *A. pyrenoidosa*.

At the physiological level, 10 mg/L tZ significantly enhanced algal cell biomass, photosynthetic pigments, and storage substances. Additionally, it reduced ROS levels and maintained redox balance by enhancing the activity of SOD and CAT, as well as promoting glutathione metabolism.

Molecular analysis revealed that tZ facilitates cellular biomass accumulation by modulating both carbon and nitrogen metabolism. Specifically, tZ improves photosynthetic efficiency by upregulating genes involved in porphyrin metabolism (*HCAR*, *UGT*, etc.), which supports Chla synthesis. It also inhibits the degradation pathways (e.g., *HEPH*) and activates key enzymes in the carbon fixation pathway (e.g., *PGK*, *GAPDH*), thereby promoting carbohydrate accumulation in algal cells. Moreover, tZ enhances nitrogen metabolism by upregulating genes such as glutamine synthetase (*GLUL*) and nitrate reductase (*NR*). This promotes the accumulation of nitrogen metabolites like arginine and polyamines, which contribute to protein synthesis and cellular growth.

These findings provide a deeper understanding of the regulatory role of cytokinins in microalgal metabolism. They also offer valuable insights for developing low-cost, high-efficiency microalgal cultivation methods, which could be beneficial for various biotechnological applications.

## Data Availability

The original contributions presented in this study are included in the article/Supplementary Material. Further inquiries can be directed to the corresponding author.

## Supplementary Materials

The following supporting information can be downloaded at: https://www.mdpi.com/article/10.3390/microorganisms13112554/s1, Table S1: Result of sample sequencing quality; Figure S1: Statistic of differentially expressed genes (DEGs): (A) Volcano Plot of DEGs; (B) Hierarchical clustering analysis of DEGs; Figure S2: Statistic of differentially expressed metabolites (DEMs): (A) Volcano Plot of DEMs; (B) Hierarchical clustering analysis of DEMs; Figure S3: Pie-chart of metabolite classification.

## Abbreviations

The following abbreviations are used in this manuscript:

SDGs	Sustainable Development Goals
*A. pyrenoidosa*	*Auxenochlorella pyrenoidosa*
tZ	trans-Zeatin
SP	soluble protein
CHO	carbohydrate
Chla	chlorophyll a
ROS	reactive oxygen species
SOD	superoxide dismutase
CAT	catalase
MDA	malondialdehyde
GSH	reduced glutathione
TAC	total antioxidant capacity
IAA	indole-3-acetic acid
GA	gibberellic acid
ABA	abscisic acid
IBA	indole-3-butyric acid
BG-11	Blue-Green 11
PBS	phosphate buffer
APX	ascorbate peroxidase
ALA	5-amino-4-oxovaleric acid
TCA	tricarboxylic acid
NADPH	Nicotinamide Adenine Dinucleotide Phosphate Reduced
DEGs	differentially expressed genes
DEMs	differentially expressed metabolites
h	hours
min	minutes
s	second
SD	standard deviation
PCA	principal component analysis
OPLS-DA	orthogonal partial least squares discriminant analysis
*GSS*	Glutathione synthetase
*GPX*	Glutathione peroxidase
*GST*	Glutathione S-transferases
*PRDX6*	Peroxiredoxin 6
*OPLAH*	5-oxoprolinase, ATP-hydrolyzing
*G6PD*	Glucose-6-phosphate dehydrogenase
*algH*	Putative transcriptional regulator
*CCT3*	T-complex protein 1 subunit gamma
*HCAR*	Heme synthase
*UGT*	UDP-glucuronosyltransferase
*HEPH*	Heme oxygenase
*sirB*	Siroheme biosynthesis
*CAO*	Chlorophyllide a oxygenase
*MDH2*	Malate dehydrogenase
*tktA*	Transketolase
*PGK*	Phosphoglycerate kinase
*rpe*	Ribulose-phosphate 3-epimerase
*TPI*	Triose-phosphate isomerase
*GAPDH*	Glyceraldehyde 3-phosphate dehydrogenase
*GLUD1_2*	Glutamate dehydrogenase (NAD(P)+)
*arcC*	Carbamate kinase
*GLUL*	Glutamine synthetase
*NR*	Nitrate reductase (NAD(P)H)
*hcp*	Hydroxylamine reductase
*cynS*	Cyanate lyase
*CYP55*	Fungal nitric oxide reductase
