# Supplementary material for: Trans-Zeatin Enhances Auxenochlorella pyrenoidosa Growth by Coordinating Carbon–Nitrogen Metabolism and Antioxidant Defense
Source: Microorganisms. 2025 Nov 8;13(11):2554. doi: 10.3390/microorganisms13112554 (PMC12654036; doi:10.3390/microorganisms13112554)
Supplement: Supplementary file 1 [file microorganisms-13-02554-s001.zip › microorganisms-3940617-supplementary.pdf]

Supplementary Materials

Table S1 Result of sample sequencing quality

Sample	Raw reads	Clean reads	Clean bases	Q20(%)	Q30(%)	GC content(%)
CK1	43443604	42475434	6.36G	98.06	94.24	63.80
CK2	45687288	44800678	6.70G	98.28	94.86	61.30
CK3	45051202	44268274	6.58G	98.20	94.77	62.89
tZ1	44288316	43532504	6.51G	98.11	94.52	63.59
tZ2	44132562	43454290	6.50G	98.18	94.70	63.13
tZ3	46005362	45265738	6.76G	98.21	94.74	63.52

Note: GC is the percentage of the total number of bases G and C in the total number of bases.

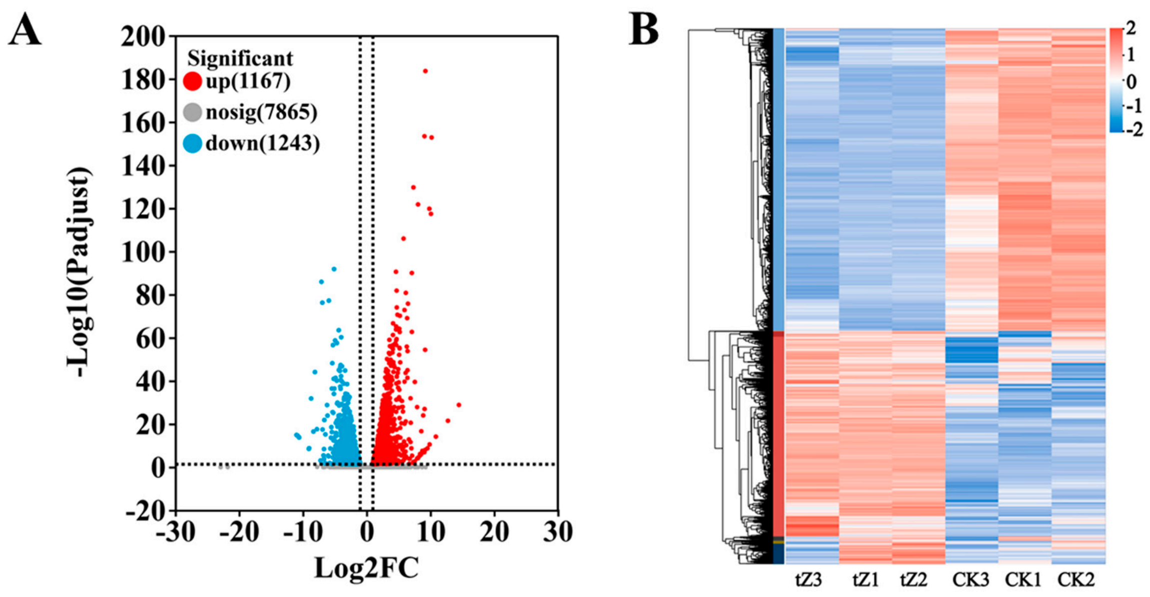

Figure S1 Statistic of differentially expressed genes (DEGs).（A）VolcanoPlot of DEGs.（B）Hierarchical clustering analysis of DEGs.

Note:In In Figure S1A, increased genes are shown in red and decreased ones in blue. Non-significant genes appear in gray.

A

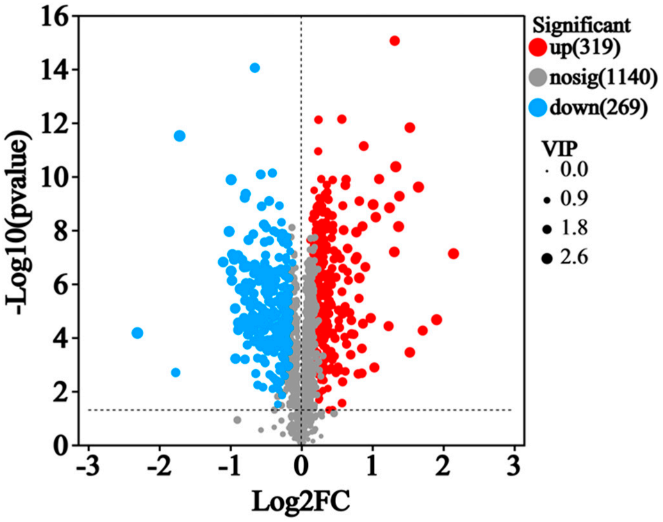

Significant
up(319)
nosig(1140)
down(269)
VIP
0.0
0.9
1.8
2.6
-Log10(pvalue)
Log2FC

B

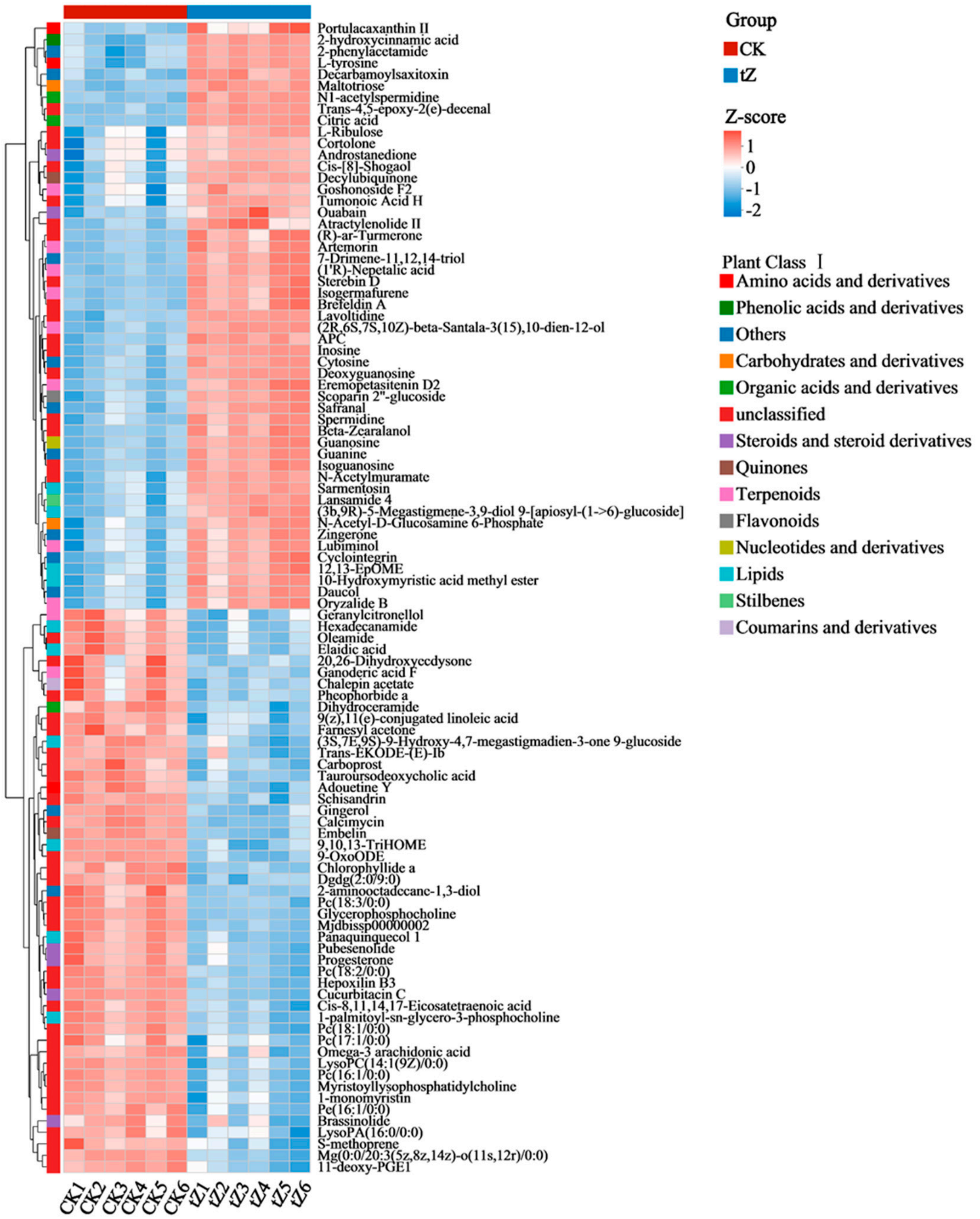

Figure S2 Statistic of differentially expressed metabolites (DEMs).（A）Volcano Plot of DEMs.（B）Hierarchical clustering analysis of DEMs.

Note:In In Figure S2A, increased metabolites are shown in red and decreased ones in blue. Non-significant metabolites appear in gray. In Figure S2B, the horizontal axis shows samples treated with different concentrations of Trans-zeatin (tZ). Higher Z-Scores indicate higher metabolite concentrations.

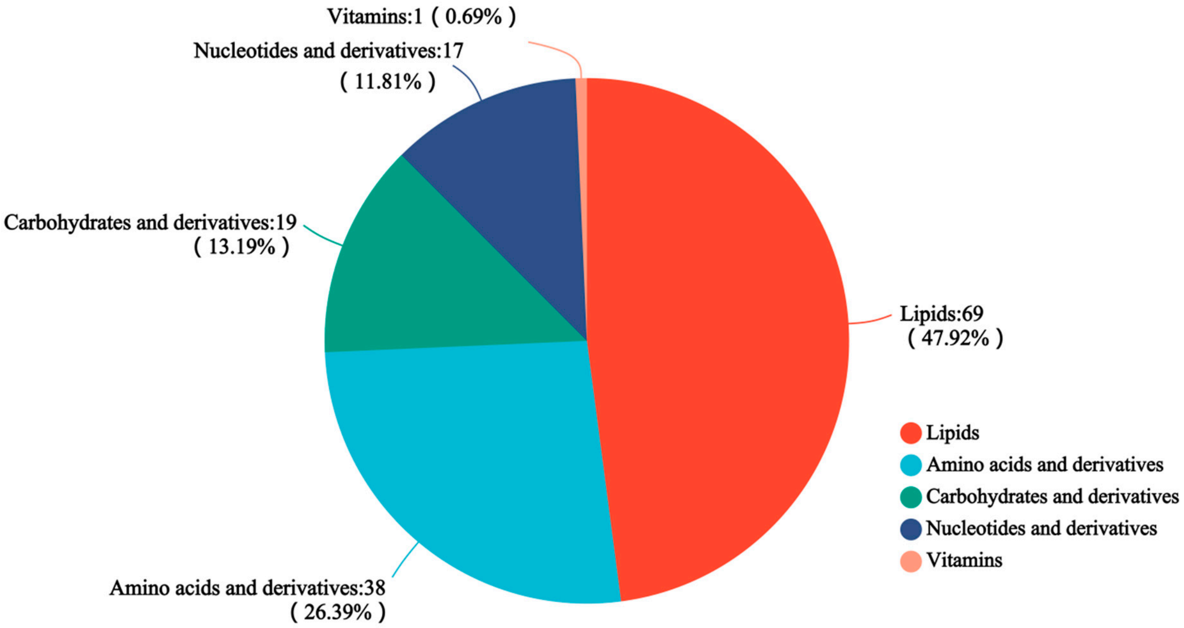

Figure S3 Pie-chart of metabolite classification
